# Coming Out to Play: Privacy, Data Protection, Children’s Health, and COVID-19 Research

**DOI:** 10.3389/fgene.2021.659027

**Published:** 2021-04-14

**Authors:** Michael J. S. Beauvais, Bartha Maria Knoppers

**Affiliations:** ^1^Centre of Genomics and Policy, Faculty of Medicine, McGill University, Montreal, QC, Canada; ^2^Canada Research Chair in Law and Medicine, Montreal, QC, Canada

**Keywords:** children, data protection, privacy, health research, pediatrics, research ethics

## Abstract

The COVID-19 pandemic has underscored the need for new ways of thinking about data protection. This is especially so in the case of health research with children. The responsible use of children’s data plays a key role in promoting children’s well-being and securing their right to health and to privacy. In this article, we contend that a contextual approach that appropriately balances children’s legal and moral rights and interests is needed when thinking about data protection issues with children. We examine three issues in health research through a child-focused lens: consent to data processing, data retention, and data protection impact assessments. We show that these issues present distinctive concerns for children and that the *General Data Protection Regulation* provides few bright-line rules. We contend that there is an opportunity for creative approaches to children’s data protection when child-specific principles, such as the best interests of the child and the child’s right to be heard, are put into dialogue with the structure and logic of data protection law.

## Introduction

It is axiomatic that children are vulnerable. Without fully formed cognitive capacities and the lack of life experience, children need help from their parents, civil society, and the State to look after their best interests. During the COVID-19 pandemic, their vulnerability as a group and as individuals has only increased. Threats to the biological existence of citizens have necessitated the use of State power to change daily social life. In the face of such changes, it is nevertheless regrettable that the rights and interests of children have been largely ignored. Indeed, for today’s children, there is a certain irony to current circumstances. By and large, children and adolescents are actually less *biologically* vulnerable to COVID-19, being spared the worst effects of the disease. Yet, where such legal and political power is leveraged in response to adult biological vulnerability, children may be pawns, owing to their political vulnerability (Larcher and Brierley, 2020).

With the COVID-19 pandemic, we are at the apex of the collection, use, and disclosure of data about children. In this article, we contend that the new ways of thinking about data protection issues with children in the health research context are overdue. We first outline the opportunities and challenges of children’s data. They are at once indispensable for the promotion of children’s rights and interests and yet pose risks to their well-being if improperly used. We then examine children’s rights to privacy and data protection under the *Convention on the Rights of the Child* (CRC; 1989) and the *General Data Protection Regulation* (GDPR; 2016). We contend that the lack of clear, child-specific provisions means that a highly contextual approach must be taken to understand the relationship of children’s privacy to health research. As such, we examine three specific issues for children and COVID-19 research: consent, data retention, and data protection impact assessments. Each of these three issues presents a delicate balancing exercise with few bright-line rules. As such, we conclude by calling for increased attention to the data protection needs of children in health research.

## Children’s Data: Opportunities and Challenges

During the pandemic, children’s lives have been transformed. Many are attending school virtually – logging on for most of the day to interact with their classmates and teachers. Even for children who are going to school in person, the management of their education has shifted dramatically. As with other infectious disease outbreaks, contact tracing is frequently used, revealing potentially sensitive information about children’s interactions with others, especially in the case of adolescents (Berman et al., 2020). It is still lively debated whether school openings are responsible for increased incidents of COVID-19 among children and adolescents, with some arguing that children are not the super spreaders many had initially worried about (Munro and Faust, 2020), and others contending that children play a key role in community transmission (Hyde, 2020). Irrespective of the validity of either hypothesis, when schools are open, consideration must be given to the allocation and prioritization of COVID-19 testing (Mathew, 2020; Pettit et al., 2020), which again generate additional data about the health status of children and their families.

Turning to the health research context, there is a wide array of questions regarding the effects of severe acute respiratory syndrome coronavirus 2 (SARS-CoV-2) on children. This ranges from concerns regarding the multisystem inflammatory syndrome in children (MIS-C) associated with COVID-19 (Consiglio et al., 2020; Jiang et al., 2020; Jones et al., 2020; Verdoni et al., 2020) to the involvement of children in clinical trials for vaccines (Anderson et al., 2020), which would eventually include on-going Phase IV monitoring (Nell, 2018), to the psychosocial toll social distancing and stay-at-home orders have had on children (Cardenas et al., 2020). This is to say nothing of the need to develop therapeutics, improve treatment protocols, and other applications of clinical knowledge in a way that attends to the specific physiological needs of children. Meeting this challenge of pandemic proportions requires the broad sharing of data among international research teams in ways that ensure children’s rights and interests are furthered and that public trust is maintained.

Big data presents both challenges and opportunities for children (Berman and Albright, 2017; Almog and Franco, 2020). Children’s rights and interests may be furthered through sophisticated analyses of data across domains – from clinical to environmental to educational. In the health context, big data in the form of -omics data has begun to show success in the stratification of sick children (Ding et al., 2019). The Committee on the Rights of the Child has encouraged States to conduct research with children “to learn about their health challenges, developmental needs, and expectations as a contribution to the design of effective interventions and health programs” (United Nations Committee on the Rights of the Child, 2013a). The Committee further opines that data concerning key health problems and health determinants should be collected through routine health information systems and through research (United Nations Committee on the Rights of the Child, 2013a).

At the same time, data may be used against children’s rights and interests through profiling, targeted advertising, and unjustified discrimination. And concerns about children’s data are growing. Parents worry about who has access to data about their children and for which purposes such data may be used (Barassi, 2020). Sociological research suggests that concerns regarding who can share what with whom are shared by both parents (Barassi, 2020; Cino and Vandini, 2020) and children (Sarkadi et al., 2020). A growing body of scholarship highlighting the potential dangers of using children’s data in ways that are not in children’s best interests accompanies these sentiments. Zuboff (2019), for example, has warned of the pitfalls of immersing children in environments that are designed to harvest data and help to shape future consumption behaviors.

## Children’s Privacy: Sources

Against this data-rich backdrop, the privacy interests of children have been hitherto underdeveloped. Enshrined in the CRC, the child’s right to privacy provides children with a right to informational privacy, as well as giving the family a sphere of decisional privacy (United Nations General Assembly, 1989). Only in the past decade or so, with the advent of social networks and other platforms that heavily rely on personal data, have children’s privacy interests garnered much interest (Dowty, 2008; Shmueli and Blecher-Prigat, 2011). The majority of scholarship and normative guidance on the topic of children’s data protection consequently have been aimed at attending to the multifaceted issues such websites present (Milkaite and Lievens, 2019). For example, the GDPR includes a special consent regime for social networking websites. Otherwise, the regulation is mostly silent on the specific issues children’s data pose, other than its express recognition that children are vulnerable. The COVID-19 pandemic has, however, spawned further research into the children’s privacy issues related to contact tracing and other public health surveillance technologies (Berman et al., 2020). Children’s privacy in health research remains nevertheless little researched.

## Best Interests of the Child

As a structuring principle for *all* children’s rights (United Nations Committee on the Rights of the Child, 2013b), the best interests of the child standard (BIC) is central to delimiting the child’s right to data protection. The BIC demands that, in all decisions concerning a child, their best interests are a primary consideration (United Nations General Assembly, 1989). That the BIC is *a* rather than *the* primary consideration means that it is not an overriding concern in all matters, i.e., it may be departed from in certain circumstances.

Despite not being children’s rights instruments *per se*, the BIC is further secured under the *European Convention of Human Rights* (ECHR; Council of Europe, 1950; European Court of Human Rights (First Section), 2007) and the *Charter of Fundamental Rights of the European Union* (CFREU; European Union, 2012). Under the ECHR, the BIC assists in the elaboration of the rights, where children are involved (Hubert-Dias, 2014). The CFREU expressly incorporates the BIC, specifying that it is a primary consideration, “in all actions relating to children, whether taken by public authorities or private institutions” (European Union, 2012, Art 24). The inclusion of the BIC in both the ECHR and CFREU is not a mere formality. The GDPR, as an elaboration of the rights to private life and to data protection under the ECHR and CFREU, uses these rights and obligations as its framework (Kuner et al., 2020). In the context of children’s data, guidance from the Article 29 Data Protection Working Part (A29 DPWP) expressly recognizes that, “the core legal principle [for data processing and beyond] is that of the best interest of the child” (Article 29 Data Protection Working Party, 2009, p. 4).

The BIC imposes an obligation on decision-makers – be they parents, policymakers, ethicists, lawyers, researchers, and others – to engage in a reasoned decision process (Eekelaar and Tobin, 2019). After having determined the best interest, any decisions to depart from what the BIC requires must be justified. The BIC further acts as an aid in interpreting and implementing the panoply of rights and obligations to which the CRC gives rise (Hammarberg, 2011). In this vein, the Committee on the Rights of the Child has recommended that the BIC guide all actions and decisions by the government concerning legislation, court decisions, administrative decisions, and projects, programs, and services that have an impact on children (United Nations Committee on the Rights of the Child, 2009). This approach requires taking into consideration a broad spectrum of factors that affect the well-being of the child (United Nations Committee on the Rights of the Child, 2013b).

Of particular import for COVID-19 research, the obligation for the BIC to be a primary consideration in all matters affecting a child includes the promotion of their health and welfare interests. In the context of research, this means that, under the broad WHO definition of “health,” their general physical and psychological well-being must be taken into account (World Health Organization, 2020). This nexus between the BIC and the inclusion of children in research as a population with specific developmental needs is accentuated in public health as there are additional implications for their future health as adults. This is all the more true when one considers that public health is founded on an ethos of supporting a public good (Upshur, 2002), which in a pandemic should include children as a vulnerable population.

The BIC also, however, acts a protective factor for research involving children. The collective societal interest in COVID-19-related does not negate the duty to ensure that any participation in research be in an individual child’s best interests or that of children of the same age or condition (World Medical Association, 2013). For example, the Committee on the Rights of the Child has stressed that the BIC requires anyone undertaking research involving child-participants to follow international ethical guidelines (United Nations Committee on the Rights of the Child, 2013a). More concretely, the Committee states categorically that the BIC “shall always prevail over the interest of general society or scientific advancement” (United Nations Committee on the Rights of the Child, 2013a, p. 85).

## Specific Issues Regarding Children’s Data Protection and Health Research

As a general proposition, then, the child’s right to data protection and to have their interest be a primary consideration is always at play. Without specific norms regarding how these rights are to be reconciled in the context of health research, we propose to look at the data protection issues that three aspects of COVID-19-related research with children present: consent, data retention, and data protection impact assessments.

### Consent

Even during a pandemic, informed ethical consent is a *sine qua non* of ethical research involving human participants. Where data processing is concerned, however, the GDPR provides various other legal bases by which personal data may be processed. Indeed, consent as a legal basis may not be appropriate in many forms of health research because of the power imbalance between the researcher-controller and the participant-data subject. Clinical trials with sick participants are one potential case (European Data Protection Board, 2019). The increased vulnerability of research with sick children only intensifies this imbalance that negates the freely given aspect of consent. If possible, scientific research related to COVID-19 may find it easier to rely upon a public interest basis for certain processing (Becker et al., 2020).

Beyond power imbalances, the complex nature of contemporary big data biomedical research stretches what one can reasonably expect data subjects to understand to give consent, especially in the case of children. This position can be inscribed in larger debates regarding the insufficiency of consent in the context of very complex data processing activities whose consequences on the data subject’s interests are difficult, if not possible, to understand (Weigend, 2017). If a competent adult hypothetically may struggle to understand the nature of processing and its consequences, conveying this to children is even more difficult. Despite these consent issues, some EU Member States have gone in the opposite direction. Ireland, for example, requires consent to be the legal basis for data processing for health research, unless certain conditions are met (Republic of Ireland, 2018).

The additional physical risks that COVID-19 creates also present difficulty, whether doing research with children or adults (Largent et al., 2020). Physical enrolment, the taking of biosamples, and other such tasks increase the risk of exposure to SARS-CoV-2. There are multiple models for mitigating these risks, such as opt-out with notification (Knoppers et al., 2020). These models may not satisfy the narrow notion of GDPR consent because, among other things, an affirmative act is required (European Data Protection Board, 2020b). Choosing another legal basis for data processing may then help to minimize contact and exposure to risk.

Findings ways to solicit the child’s views and give effect to them in these circumstances must be carefully considered. Parents or other legally authorized representatives exercise rights on behalf of children, and thus are the only ones who may give a legally valid consent, saving a judicial order specifying otherwise. There is, however, a dynamic process between children and parents in giving effect to a child’s burgeoning autonomy. Under the CRC, children have a right to be heard (United Nations General Assembly, 1989). Giving effect to this right requires that there be opportunity for the child to make their views known and that any decisions be justified in light of these views.

So-called “mature minor” doctrines are aimed at giving effect to a child’s views when they understand the nature and consequences of a procedure and the procedure is viewed to be in that child’s best interests (Appellate Committee of the House of Lords, 1985; Dalpé et al., 2019). Some have argued that the mature minor doctrine should be transposed into the data protection context for children (Buitelaar, 2018). However, transposing such a contextually specific doctrine to health research, and to data processing more generally, raises more questions than answers (Taylor et al., 2017). Clinical decision-making implies a different range of considerations than in health research and in data processing, e.g., the expectation that the procedure is likely to confer health benefits upon the patient, clinical procedures involve a child’s physical integrity, etc.

To take the child’s autonomy seriously, it has been suggested that data controllers have ongoing, transparent engagement with the child data subjects (Taylor et al., 2017). Such an approach may be particularly well suited for biobanking and other such longitudinal studies. For research projects with shorter timescales and less resources for participant engagement, this could pose challenges. A project webpage that includes age-appropriate consent and assent materials may go a long way to ensuring that parents and children are sufficiently engaged.

### Data Retention (Storage)

The diverse array of host genomic and phenotypic data collected during the course of COVID-19-related research may be met with an uncertain future. The EDPB’s own guidelines for scientific research during the COVID-19 pandemic state categorically that “storage periods (timelines) shall be set and must be proportionate” (European Data Protection Board, 2020a, p. 13). Paradoxically, however, we note that the GDPR is clear on this point: personal data used exclusively for research purposes may be kept indefinitely, provided that there are appropriate safeguards in place (Bovenberg et al., 2020). Any secondary use would then be limited to other scientific research studies (or for archiving in the public interest and historical research or statistical purposes). Although this does not confer unfettered discretion on researcher-controllers, it should allow for undefined storage periods in the case of data that are either difficult to generate or even impossible to generate again because they represent the child’s health indicators at a given moment in time.

Children, again, hypothetically pose specific issues. And, perhaps unsurprisingly, the EDPB’s COVID-19 scientific research guidelines are silent on this point. Due to the physiological changes that children undergo, it is more likely that certain health data represent unique points in time and cannot be replicated. This justification must be weighed against the potential risks that continued storage presents to the children-data subjects. Depending on the research type and the data generated, this may include discrimination, embarrassment, or other social stigma in the case of a data breach. In other words, utility must confront vulnerability.

Taking the BIC seriously as regards data retention would suggest that less data be retained, unless retention can be justified to serve an objective or interest that supersedes the child’s best interests. Given that the A29 DPWP has taken an expansive approach to the notion of data subject interests that goes beyond legal interests, ethical principles, and concerns may also hypothetically feature in the analysis (Article 29 Data Protection Working Party, 2014). This approach could accommodate non-legal notions such as the child’s moral “right to an open future” (Feinberg, 1980).

The child’s moral right to an open future is a complex consideration for data storage, and indeed data processing writ large. At its core, the right calls for parents to conserve certain decisions for children when such decisions may be made autonomously by the child. This would seem to militate against data retention. Nevertheless, autonomy may still work with data retention. Providing the child with, and facilitating the exercise of, the ability to opt-out at the age of majority, discussed below, may also be seen as compatible with the child’s moral right to an open future. The moral right to an open future should, in our estimation, also feature as a concern of data minimization, thus decreasing risks to child data subjects.

Assuming that data are retained: the numerous research studies involving children and COVID-19 will eventually have to confront another reality: what happens with data when the child-participants reach the age of majority? According to A29 DPWP guidance, where consent is the legal basis for processing, it is unlikely that the parental consent alone will be sufficient to justify continued processing once the child reaches the age of majority (Article 29 Data Protection Working Party, 2009). Where consent is not the legal basis relied upon, the issue is very open textured. In keeping with the core principles of transparency and accountability, controller-researchers should strive, at a minimum, to notify participants about their data and the research when they reach the age of majority and provide the opportunity for opt-out. This allows the newly emancipated participants the ability to decide what is done with their data in a way that strikes a balance with the enduring interest in health research. Such an approach further coheres with the choice within the GDPR to include a right to object, for instances, where consent is not the legal basis for processing.

### Data Protection Impact Assessments

At every turn thus far, we have advocated for a balancing exercise when it comes to the contextual nature of children’s data protection in the health context. Whether issues relate to giving due respect to the child’s best interests, child and parental autonomy, or data retention, child-data subjects present distinct concerns for which few bright-line norms exist. Indeed, analyzing what the broad spectrum of rights secured under the CRC requires in any context implicates a weighing exercise.

One particularly germane tool for this weighing exercise is a data protection impact assessment (DPIA; van der Hof and Lievens, 2018). DPIAs are a tool to analyze the scope and effects of data processing, where processing is “likely to result in a high risk to the rights and freedoms of natural persons.” DPIAs present the opportunity for controller-researchers to carefully examine the risks that inhere to data processing throughout its lifecycle and to then implement safeguards to reduce or eliminate such risks. Because DPIAs are meant to be conducted from the point-of-view of the data subject (Article 29 Data Protection Working Party, 2017), they lend themselves to engaging with children to understand the risks certain scientific data processing tasks may pose to their interests.

As a tool that is meant to be updated as processing operations change, it can be updated in response to the evolving capacities of children for longitudinal studies. Making the DPIAs available to parents and child-participants would do much to further transparency, accountability, and trust. In the case of presenting a DPIA to children, it should be tailored to their level of understanding (Lievens and Verdoodt, 2018). If properly done, a DPIA may be useful for seeking informed consent to research (ethics consent), or for even teaching children (and parents) about the risks and benefits of data processing and how the researcher-controller is keeping their data secure, helping to create the “tripartite relationship of mutual trust between patients, families and health care teams” that pediatric data sharing requires (Rahimzadeh et al., 2018, p. 477).

## Conclusion

The COVID-19 pandemic has put our normative frameworks to the test in many regards. For children’s rights, perhaps the most difficult has been ensuring that these rights and the interests that ground them are taken into account when it comes to crafting public health measures. Beyond immediate public health concerns, we have seen a phenomenal expansion of children’s digital footprints. Although much of this has happened outside of the health research context through changes, such as online schooling and increased reliance on digital technologies for socializing, the changes emphasize the need to think more about children’s right to data protection and its interaction with other children’s rights, in particular the right to health. Science may be able to bring the pandemic to an end, but it cannot answer important normative questions such as those that relate to children’s data.

We have striven to canvas issues with a pragmatic lens to real-world issues that COVID-19 research with children may present. Yet, we have seen that there is little authoritative guidance regarding data protection law and its application to children. On the one hand, this is an opportunity; the silence of norms invites creative thinking and flexibility for researcher-controllers and policymakers. On the other hand, researcher-controllers are forced to confront potentially difficult choices to which even the best of intentions may not quickly provide an answer. At minimum, though, the need to take into account children’s best interests and to provide reasoned justification in a transparent manner are central to any effective approach to children’s data protection. Further elucidating issues related to children’s data protection as regards their health data should be a central concern of scholars, researchers, policy makers, and clinicians alike.

## Data Availability Statement

The original contributions presented in the study are included in the article/supplementary material, further inquiries can be directed to the corresponding author.

## Author Contributions

MB and BK conceived the article. MB conducted the research and drafting in consultation with BK. All authors contributed to the article and approved the submitted version.

### Conflict of Interest

The authors declare that the research was conducted in the absence of any commercial or financial relationships that could be construed as a potential conflict of interest.
